# Unintentional Firearm Injury Deaths Among Children and Adolescents Aged 0–17 Years — National Violent Death Reporting System, United States, 2003–2021

**DOI:** 10.15585/mmwr.mm7250a1

**Published:** 2023-12-15

**Authors:** Rebecca F. Wilson, Sasha Mintz, Janet M. Blair, Carter J. Betz, Abby Collier, Katherine A. Fowler

**Affiliations:** ^1^Division of Violence Prevention, National Center for Injury Prevention and Control, CDC; ^2^National Center for Fatality Review and Prevention, Michigan Public Health Institute, Okemos, Michigan.

**Summary**

**What is already known about this topic?**

Unintentional injury is a leading cause of death among U.S. children and adolescents aged 0–17 years, and firearms are a leading injury method.

**What is added by this report?**

Approximately one half of unintentional firearm injury deaths among children and adolescents occurred at their home; playing with or showing the firearm to another person was the most common precipitator. Overall, firearms used in unintentional injury deaths were often stored both loaded and unlocked and were commonly accessed from nightstands and other sleeping areas.

**What are the implications for public health practice?**

Unintentional firearm injury deaths are preventable. Securing firearms (e.g., locked, unloaded, and separate from ammunition) is protective against unintentional firearm injury deaths among children and adolescents, underscoring the importance of promoting secure firearm storage.

## Abstract

In the United States, unintentional injury is the fourth leading cause of death among infants (i.e., children aged <1 year) and is the top cause of death among children and adolescents aged 1–17 years; firearms are a leading injury method. Unsecured firearms (e.g., unlocked and loaded) are associated with risk for unintentional childhood firearm injury death. Data recorded during 2003–2021 by the National Violent Death Reporting System (NVDRS) from 49 states, the District of Columbia, and Puerto Rico were used to characterize unintentional firearm injury deaths of U.S. infants, children, and adolescents aged 0–17 years (referred to as children in this report). NVDRS identified 1,262 unintentional firearm injury deaths among children aged 0–17 years: the largest percentage (33%) of these deaths were among children aged 11–15 years, followed by 29% among those aged 0–5 years, 24% among those aged 16–17 years, and 14% among persons aged 6–10 years. Overall, 83% of unintentional firearm injury deaths occurred among boys. The majority (85%) of victims were fatally injured at a house or apartment, including 56% in their own home. Approximately one half (53%) of fatal unintentional firearm injuries to children were inflicted by others; 38% were self-inflicted. In 9% of incidents, it was unknown whether the injury was self- or other-inflicted. Approximately two thirds (67%) of shooters were playing with or showing the firearm to others when it discharged. Overall, firearms used in unintentional injury deaths were often stored loaded (74%) and unlocked (76%) and were most commonly accessed from nightstands and other sleeping areas (30%). Unintentional firearm injury deaths of children are preventable. Secured firearm storage practices (e.g., storing firearms locked, unloaded, and separate from ammunition) have been identified as protective factors against child firearm injuries and deaths, underscoring the importance of policymakers, health care professionals (e.g., pediatricians), and others partnering with parents, caregivers, and firearm owners to promote secure firearm storage.

## Introduction

In the United States, unintentional injury is the fourth leading cause of death among infants (i.e., children aged <1 year) and is the top cause of death among children and adolescents aged 1–17 years; firearms are a leading injury method.* Unsecure firearm storage practices (e.g., storing firearms unlocked and loaded) are associated with risk for unintentional and intentional (i.e., suicide) firearm injuries and deaths among children and adolescents (*1*). Most unintentional firearm injuries among children occur within the home, with firearms predominantly originating in the child’s home (*1*,*2*). In 2021, approximately 30 million children lived in homes with firearms, including 4.6 million in households reporting storing firearms loaded and unlocked (*3*). CDC analyzed unintentional firearm injury deaths among infants, children, adolescents aged 0–17 years (referred to as children in this report) in the United States to examine their characteristics.

## Methods

Data from CDC’s National Violent Death Reporting System (NVDRS) for 49 states, District of Columbia, and Puerto Rico^†^ for 2003–2021 were analyzed. NVDRS is a state-based active surveillance system linking information from death certificates, law enforcement reports, and coroner or medical examiner records into one incident. Trained abstractors enter information into a web-based system using standardized coding guidance from CDC. Additional details on methodology are available from NVDRS.^§^ An unintentional firearm injury death is defined in NVDRS as one “resulting from a penetrating injury or gunshot wound from a weapon that uses a powder charge to fire a projectile^¶^ when there was a preponderance of evidence that the shooting was not intentionally directed at the victim.” Fatal unintentional firearm injury cases for this study were identified based on the following manners of death: 1) unintentional firearm injury deaths, 2) homicides, and 3) undetermined intent deaths. *International Classification of Diseases, Tenth Revision* underlying cause of death codes W32, W33, W34, and Y86 were used to identify potential cases. Cases were excluded if the method of injury was a nonfirearm weapon type or nonpowder BB or pellet gun, or if the case did not meet NVDRS case definition for an unintentional firearm injury death. Age categories used in this study were selected based on the age-related risk of fatal unintentional firearm injury in children.** A qualitative review of free text fields (e.g., law enforcement narratives) was completed by two reviewers to enhance completeness and accuracy of data. Each reviewer assessed a random sample of 5% of the other reviewer’s cases, reconciling all discrepancies until 100% agreement was reached. Descriptive analyses were conducted using SAS (version 9.4; SAS Institute). This activity was reviewed by CDC, deemed not research, and was conducted consistent with applicable federal law and CDC policy.^††^

## Results

### Sex, Age Group, and Race and Ethnicity of Decedents

During 2003–2021, a total of 1,262 fatal unintentional firearm injury cases^§§^ among children aged 0–17 years were identified in NVDRS (Figure). A majority (83.1%) of these deaths occurred among boys (Table 1). Children aged 0–5 years accounted for 29.1% of unintentional firearm injury deaths, followed by those aged 6–10 years (14.0%), 11–15 years (33.0%), and 16–17 years (23.9%). A majority of victims were in one of the following three racial and ethnic groups: non-Hispanic Black or African American (39.9%), Hispanic or Latino (10.7%), and non-Hispanic White (42.2%).

**FIGURE:**
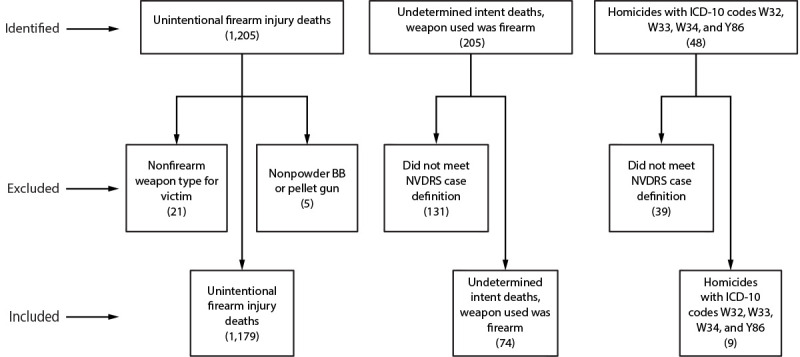
Identification of unintentional firearm injury deaths among children and adolescents aged 0–17 years (N = 1,262) — National Violent Death Reporting System, United States, 2003–2021* **Abbreviations**: ICD-10 = *International Classification of Diseases, Tenth Revision*; NVDRS = National Violent Death Reporting System. * The final sample of included cases comprised 93.4% of unintentional firearm injury deaths as identified by NVDRS; 5.9% undetermined intent deaths by firearm; and 0.7% homicides with ICD-10 codes W32, W33, W34, and Y86 bringing the total case count of unintentional firearm injury deaths to 1,262.

**TABLE 1:** Number and percentage* of unintentional firearm injury deaths among children and adolescents aged 0–17 years, by victim’s sex and race and ethnicity; shooter; and shooter’s age, sex, and relationship to the victim (N = 1,262) — National Violent Death Reporting System, United States,^†^ 2003–2021

Characteristic	Victim age group,^§^ yrs, no. (column %*)				
	0–5	6–10	11–15	16–17	Total
**Victim sex**					
Boy	285 (77.7)	134 (76.1)	361 (86.6)	269 (89.1)	**1,049 (83.1)**
Girl	82 (22.3)	42 (23.9)	56 (13.4)	33 (10.9)	**213 (16.9)**
**Total (row %)**	**367 (29.1)**	**176 (14.0)**	**417 (33.0)**	**302 (23.9)**	**1,262 (100.0)**
**Victim race and ethnicity**					
A/PI	5 (1.4)	1 (0.6)	2 (0.5)	2 (0.7)	**10 (0.8)**
AI/AN	6 (1.6)	11 (6.3)	17 (4.1)	13 (4.3)	**47 (3.7)**
Black or African American	179 (48.8)	78 (44.3)	133 (31.9)	113 (37.4)	**503 (39.9)**
White	137 (37.3)	67 (38.1)	206 (49.4)	122 (40.4)	**532 (42.2)**
Hispanic or Latino^¶^	24 (6.5)	18 (10.2)	49 (11.8)	44 (14.6)	**135 (10.7)**
Two or more, other, unspecified race	16 (4.4)	1 (0.6)	10 (2.4)	8 (2.7)	**35 (2.8)**
**Total (row %)**	**367 (29.1)**	**176 (14.0)**	**417 (33.0)**	**302 (23.9)**	**1,262 (100.0)**
Shooter**					
Self-inflicted	212 (57.8)	44 (25.0)	122 (29.3)	99 (32.8)	**477 (37.8)**
Inflicted by other person	117 (31.9)	113 (64.2)	256 (61.4)	181 (60.0)	**667 (52.9)**
Unknown who inflicted	38 (10.4)	19 (10.8)	39 (9.4)	22 (7.3)	**118 (9.4)**
**Total (row %)**	**367 (29.1)**	**176 (14.0)**	**417 (33.0)**	**302 (23.9)**	**1,262 (100.0)**
**Shooter age when injuries inflicted by another, age group, yrs**					
2–5	35 (37.2)	10 (11.0)	3 (1.5)	0 (—)	**48 (9.5)**
6–10	27 (28.7)	21 (23.1)	14 (6.9)	1 (0.8)	**63 (12.4)**
11–17	8 (8.5)	45 (49.5)	146 (72.3)	71 (59.2)	**270 (53.3)**
≥18	24 (25.5)	15 (16.5)	39 (19.3)	48 (40.0)	**126 (24.9)**
**Total (row %) **	**94 (18.5)**	**91 (18.0)**	**202 (39.8)**	**120 (23.7)**	**507 (100.0)**
**Shooter sex when injuries inflicted by another**					
Female	9 (9.2)	6 (5.8)	10 (4.3)	11 (7.1)	**36 (6.1)**
Male	89 (90.8)	97 (94.2)	223 (95.7)	144 (93.0)	**553 (93.9)**
**Total (row %)**	**98 (16.6)**	**103 (17.5)**	**233 (39.6)**	**155 (26.3)**	**589 (100.0)**
**Shooter relationship to victim**					
Acquaintance or friend	2 (2.0)	9 (8.8)	132 (55.2)	104 (68.9)	**247 (41.7)**
Sibling	59 (59.0)	55 (53.9)	57 (23.9)	17 (11.3)	**188 (31.8)**
Other relative (e.g., cousin or uncle)	11 (11.0)	22 (21.6)	24 (10.0)	13 (8.6)	**70 (11.8)**
Parent	22 (22.0)	10 (9.8)	10 (4.2)	1 (0.7)	**43 (7.3)**
Other person known to victim (e.g., babysitter)	5 (5.0)	5 (4.9)	8 (3.4)	5 (3.3)	**23 (3.9)**
Current dating partner	0 (—)	0 (—)	4 (1.7)	10 (6.6)	**14 (2.4)**
Other (e.g., stranger)	1 (1.0)	1 (1.0)	4 (1.7)	1 (0.7)	**7 (1.2)**
**Total (row %)**	**100 (16.9)**	**102 (17.2)**	**239 (40.4)**	**151 (25.5)**	**592 (100.0)**

**Abbreviations:** AI/AN = American Indian or Alaska Native; A/PI = Asian or Pacific Islander.

* Percentages might not sum to 100 because of rounding. Except where noted, the denominator excluded those unintentional firearm injury deaths where characteristics were missing or unknown. Percentages are column percentages, unless otherwise noted.

^†^ Data from 49 states, District of Columbia, and Puerto Rico that reported information to the National Violent Death Reporting System during 2003–2021 were included in this analysis. These jurisdictions included Alaska, Maryland, Massachusetts, New Jersey, Oregon, South Carolina, and Virginia (2003–2021); Colorado, Georgia, North Carolina, Oklahoma, Rhode Island, and Wisconsin (2004–2021); Kentucky, New Mexico, and Utah (2005–2021); Ohio (2011–2021); Michigan (2014–2021); New York (2015–2018 and 2020–2021); Hawaii (2015, 2016, and 2019); Arizona, Connecticut, Kansas, Maine, Minnesota, New Hampshire, and Vermont (2015–2021); Illinois, Indiana, Iowa, Pennsylvania, and Washington (2016–2021); California, Delaware, District of Columbia, Nevada, Puerto Rico, and West Virginia (2017–2021); Alabama, Louisiana, Missouri, and Nebraska (2018–2021); Montana, North Dakota, and Wyoming (2019–2021); and Arkansas, Idaho, Mississippi, South Dakota, Tennessee, and Texas (2021).

^§^ Age categories were selected based on the age-related risk for fatal unintentional firearm injury in children.

^¶^ Hispanic or Latino (Hispanic) persons might be of any race but were categorized as Hispanic; all racial groups were non-Hispanic.

** Coroner or medical examiner and law enforcement narratives were reviewed to enhance data completeness and accuracy of this variable.

### Shooter Characteristics in Unintentional Firearm Injury Deaths Among Children

Approximately one half (52.9%) of fatal unintentional firearm injuries among children were inflicted by another person; this proportion was highest among victims aged 6–10 years (64.2%), followed by those aged 11–15 years (61.4%), and aged 16–17 years (60.0%). Self-inflicted injuries accounted for 37.8% of childhood unintentional firearm injury deaths overall; the proportion of injuries that were self-inflicted was highest among children aged 0–5 years (57.8%). In 9.4% of incidents, it was unknown whether the injury was self-or other-inflicted (Table 1). Among fatal unintentional firearm injuries inflicted by another, in those cases for which the sex and age of the shooter were known, 93.9% of shooters were male and 75.2% were aged 2–17 years. Most shooters of children aged 6–10, 11–15, and 16–17 years were aged 11–17 years (63.4%), whereas children aged 0–5 years were most frequently shot by another child in their own age group (37.2%). When the shooter’s relationship to the child victim was known, 41.7% of victims were shot by a friend or acquaintance (including 55.2% of victims aged 11–15 years and 68.9% of victims aged 16–17 years); 31.8% of victims were shot by a sibling (including 59.0% of victims aged 0–5 years and 53.9% of victims aged 6–10 years); 11.8% were shot by another relative (e.g., cousin); and 7.3% were shot by a parent.

### Location of Injury, Precipitating Circumstances, and Incident Characteristics

The majority (85.5%) of victims were fatally injured at a house or apartment, including 55.6% in their own home (Table 2). Among all child victims of unintentional fatal firearm injuries, the most common precipitating circumstances were the shooter playing with or showing the firearm to another person (66.6%); unintentionally pulling the trigger (21.3%); thinking the firearm was unloaded, the safety was engaged, or the magazine was disengaged (20.5%); and mistaking the firearm for a toy (10.6%; most commonly among children aged 0–5 years [28.0%]) (Table 2). In approximately one third (34.1%) of all incidents, another child or other children were present or witnessed the fatal event. Nearly one half (44.6%) of firearms inflicting the fatal injury belonged to a parent of the shooter. Among incidents with known storage information, firearms used to inflict the fatal injury were stored loaded and unlocked in 73.8% and 76.2% of incidents, respectively. Among firearms that were stored unlocked and for which loaded status was known, 90.6% were stored loaded. When stored unlocked, the most common places from which the firearm was accessed were inside or on top of a nightstand, under a mattress or bed pillow, or on top of a bed (30.0%); on top of a shelf or inside a closet (18.6%); and inside a vehicle (12.5%). Handguns accounted for 74.0% of firearms used in unintentional firearm injury deaths of children.

**TABLE 2:** Number and percentage* of unintentional firearm injury deaths of children and adolescents aged 0–17 years, by precipitating circumstances and incident characteristics (N = 1,262) — National Violent Death Reporting System, United States,^†^ 2003–2021

Precipitating circumstance and incident characteristic	Victim’s age group, yrs, no. (column %)^§^				
	0–5	6–10	11–15	16–17	Total
**Precipitating circumstance** ^¶^	**311 (27.8)**	**156 (13.9)**	**380 (33.9)**	**273 (24.4)**	**1,120 (100.0)**
Playing with firearm, showing firearm to others	207 (66.6)	102 (65.4)	255 (67.1)	182 (66.7)	**746 (66.6)**
Unintentionally pulled trigger	61 (19.6)	29 (18.6)	88 (23.2)	60 (22.0)	**238 (21.3)**
Thought firearm was unloaded, safety engaged, or magazine disengaged	17 (5.5)	26 (16.7)	113 (29.7)	74 (27.1)	**230 (20.5)**
Firearm mistaken for a toy	87 (28.0)	24 (15.4)	7 (1.8)	1 (0.4)	**119 (10.6)**
Hunting or target shooting	3 (1.0)	16 (10.3)	46 (12.1)	20 (7.3)	**85 (7.6)**
Firearm was defective or malfunctioned or was fired when dropped, holstering, or operating safety lock	12 (3.9)	12 (7.7)	32 (8.4)	24 (8.8)	**80 (7.1)**
Firearm fired while loading, unloading, or cleaning	9 (2.9)	12 (7.7)	35 (9.2)	22 (8.1)	**78 (7.0)**
Other context of injury or mechanism of injury (e.g., celebratory firing of firearm)	90 (28.9)	38 (24.4)	97 (25.5)	74 (27.1)	**299 (26.7)**
**Incident characteristic**					
Firearm storage (locked status)**					
Unlocked	252 (95.5)	76 (73.1)	129 (61.4)	51 (57.3)	**508 (76.2)**
Locked	3 (1.1)	7 (6.7)	19 (9.1)	5 (5.6)	**34 (5.1)**
NA (e.g., firearm discharged during hunting)**	9 (3.4)	21 (20.2)	62 (29.5)	33 (37.1)	**125 (18.7)**
**Total (row %)**	**264 (39.6)**	**104 (15.6)**	**210 (31.5)**	**89 (13.3)**	**667 (100.0)**
Firearm storage (loaded status)**					
Loaded	199 (96.6)	67 (68.4)	111 (59.7)	51 (56.7)	**428 (73.8)**
Unloaded	0 (—)	12 (12.2)	11 (5.9)	5 (5.6)	**28 (4.8)**
NA (e.g., firearm discharged during hunting)**	7 (3.4)	19 (19.4)	64 (34.4)	34 (37.8)	**124 (21.4)**
**Total (row %)**	**206 (35.5)**	**98 (16.9)**	**186 (32.1)**	**90 (15.5)**	**580 (100.0)**
**Firearm storage (both loaded and unlocked status)^††^**					
Loaded and unlocked	190 (99.0)	57 (81.4)	87 (82.1)	43 (89.6)	**377 (90.6)**
**Total (row %)**	**192 (46.2)**	**70 (16.8)**	**106 (25.5)**	**48 (11.5)**	**416 (100.0)**
**Owner of firearm used in the fatal event**					
Parent of the shooter	125 (60.4)	41 (47.1)	66 (36.7)	16 (19.5)	**248 (44.6)**
Shooter	13 (6.3)	13 (14.9)	33 (18.3)	42 (51.2)	**101 (18.2)**
Other family member (e.g., cousin)	44 (21.3)	17 (19.5)	28 (15.6)	4 (4.9)	**93 (16.7)**
Friend or acquaintance	6 (2.9)	7 (8.1)	17 (9.4)	8 (9.8)	**38 (6.8)**
Stranger	1 (0.5)	2 (2.3)	8 (4.4)	4 (4.9)	**15 (2.7)**
Other (e.g., mother’s boyfriend)	18 (8.7)	7 (8.1)	28 (15.6)	8 (9.8)	**61 (11.0)**
**Total (row %)**	**207 (37.2)**	**87 (15.7)**	**180 (32.4)**	**82 (14.8)**	**556 (100.0)**
**Location where shooter accessed firearm used in fatal event^§§^**					
Inside or on top of nightstand, under mattress or pillow, or on top of bed	70 (33.5)	15 (26.8)	22 (25.3)	6 (24.0)	**113 (30.0)**
On top of shelf or inside a closet	30 (14.4)	17 (30.4)	20 (23.0)	3 (12.0)	**70 (18.6)**
Inside a vehicle	25 (12.0)	6 (10.7)	9 (10.3)	7 (28.0)	**47 (12.5)**
Inside a handbag, backpack, gym bag, purse, or clothing	16 (7.7)	4 (7.1)	9 (10.3)	4 (16.0)	**33 (8.8)**
On top of coffee table, kitchen table, refrigerator, or inside kitchen drawer	20 (9.6)	3 (5.4)	7 (8.1)	2 (8.0)	**32 (8.5)**
Inside room in house (unspecified where in room, but firearm stored unlocked)	13 (6.2)	6 (10.7)	5 (5.8)	1 (4.0)	**25 (6.6)**
Under couch, chair, or couch pillow	17 (8.1)	1 (1.8)	1 (1.2)	0 (—)	**19 (5.0)**
Behind furniture or leaning against something	9 (4.3)	1 (1.8)	6 (6.9)	1 (4.0)	**17 (4.5)**
Other container (e.g., shoebox or Tupperware)	9 (4.3)	1 (1.8)	3 (3.5)	0 (—)	**13 (3.5)**
Other location (e.g., inside a shed)	0 (—)	2 (3.6)	5 (5.8)	1 (4.0)	**8 (2.1)**
**Total (row %)**	**209 (55.4)**	**56 (14.9)**	**87 (23.1)**	**25 (6.6)**	**377 (100.0)**
**Location where Injury occurred **					
House or apartment	328 (92.9)	146 (85.9)	335 (82.5)	228 (80.3)	**1,037 (85.5)**
Natural area	4 (1.1)	14 (8.2)	33 (8.1)	15 (5.3)	**66 (5.4)**
Motor vehicle	12 (3.4)	4 (2.4)	11 (2.7)	16 (5.6)	**43 (3.5)**
Other location	9 (2.6)	6 (3.5)	27 (6.7)	25 (8.8)	**67 (5.5)**
**Total (row %)**	**353 (29.1)**	**170 (14.0)**	**406 (33.5)**	**284 (23.4)**	**1,213 (100.0)**
**Event characteristic **					
Injured at victim’s home	259 (75.1)	95 (58.3)	190 (47.6)	112 (41.0)	**656 (55.6)**
**Total (row %)**	**345 (29.2)**	**163 (13.8)**	**399 (33.8)**	**273 (23.1)**	**1,180 (100.0)**
**Stolen firearm**					
Firearm used to inflict fatal injury was listed or reported as stolen	11 (7.3)	1 (1.4)	23 (13.6)	24 (25.5)	**59 (12.2)**
**Total (row %)**	**150 (31.0)**	**71 (14.7)**	**169 (34.9)**	**94 (19.4)**	**484 (100.0)**
**Violence exposure**					
Other child or children present or witnessed fatal incident^¶¶^	128 (34.9)	82 (46.6)	145 (34.8)	75 (24.8)	**430 (34.1)**
**Total (row %)**	**367 (29.1)**	**176 (14.0)**	**417 (33.0)**	**302 (23.9)**	**1,262 (100.0)**
**Type of firearm used in fatal event**					
Handgun	265 (88.6)	94 (64.0)	236 (65.4)	184 (74.8)	**779 (74.0)**
Long gun (i.e., rifles, shotguns, or miscellaneous long guns) other	34 (11.4)	53 (36.1)	125 (34.6)	62 (25.2)	**274 (26.0)**
**Total (row %)**	**299 (28.4)**	**147 (14.0)**	**361 (34.3)**	**246 (23.4)**	**1,053 (100.0)**

**Abbreviations**: NA = not applicable; NVDRS = National Violent Death Reporting System.

* Percentages might not sum to 100% because of rounding. Except where noted, the denominator excluded those unintentional firearm injury deaths where characteristics were missing or unknown. Percentages are column percentages unless otherwise noted.

**^†^** Data from 49 states, District of Columbia, and Puerto Rico that reported information to NVDRS during 2003–2021 were included in this analysis. These jurisdictions included Alaska, Maryland, Massachusetts, New Jersey, Oregon, South Carolina, and Virginia (2003–2021); Colorado, Georgia, North Carolina, Oklahoma, Rhode Island, and Wisconsin (2004–2021); Kentucky, New Mexico, and Utah (2005–2021); Ohio (2011–2021); Michigan (2014–2021); New York (2015–2018 and 2020–2021); Hawaii (2015, 2016, and 2019); Arizona, Connecticut, Kansas, Maine, Minnesota, New Hampshire, and Vermont (2015–2021); Illinois, Indiana, Iowa, Pennsylvania, and Washington (2016–2021); California, Delaware, District of Columbia, Nevada, Puerto Rico, and West Virginia (2017–2021); Alabama, Louisiana, Missouri, and Nebraska (2018–2021); Montana, North Dakota, and Wyoming (2019–2021); and Arkansas, Idaho, Mississippi, South Dakota, Tennessee, and Texas (2021).

^§^ Age categories were selected based on the age-related risk for fatal unintentional firearm injury among children.

^¶^ Includes unintentional firearm injury deaths with one or more precipitating circumstances; circumstances were unknown in 142 incidents. Total numbers do not equal the sums of the columns because more than one circumstance could have been present per decedent.

** Coroner or medical examiner and law enforcement narratives were reviewed to enhance data completeness and accuracy of this variable. An example of NA is when a child died from firearm-related injuries during a hunting-related accident or target shooting, etc.; therefore, the firearm was loaded, unlocked, or loaded and unlocked due to active use.

**^††^** Denominator excludes incidents where firearm stored locked or loaded was NA (e.g., firearm discharged during hunting or target shooting). Among firearms that were stored unlocked and for which loaded status was known, 90.6% were stored loaded. Among firearms that were stored unlocked and for which loaded status was known, 9.4% were stored unloaded.

^§§^ A free-text field called “gun access narrative” is collected in NVDRS to characterize where and from whom firearms used in deaths in the system were obtained. Broad categories were created to categorize information regarding location from which the firearm was accessed for purposes of this study. Coroner or medical examiner and law enforcement narratives were reviewed to enhance data completeness and accuracy of this variable. Denominator includes incidents during which firearm access information was known and firearm was stored unlocked.

^¶¶^ “Other child(ren) present and or witnessed the fatal incident” circumstance has been collected in NVDRS for decedents since data year 2019. Coroner or medical examiner and law enforcement narratives were reviewed for 2003–2018 for all unintentional firearm injury deaths of children aged 0–17 years to enhance completeness of this variable for all years of the study. Guidance used to code this variable was obtained from pages 63–64 of the NVDRS coding manual version 6.0 (https://www.cdc.gov/violenceprevention/pdf/nvdrs/nvdrsCodingManual.pdf). The denominator for this variable included those unintentional firearm injury deaths where “Other child(ren) present and or witnessed” was missing and unknown.

## Discussion

In this analysis, most unintentional firearm injury deaths among children occurred in homes, and firearms used most often belonged to the parent of the shooter. Approximately one half of fatal injuries were inflicted by others, but consistent with other studies (*4*), injuries to children aged 0–5 years were disproportionately self-inflicted. Firearms used to inflict fatal unintentional injuries were frequently stored both loaded and unlocked, factors that have previously been associated with firearm injuries and deaths of children (*1*). These results underscore the importance of 1) promoting secure firearm storage practices (e.g., storing firearms locked, unloaded, and separate from ammunition) and parental supervision (especially when a firearm is in the home), and 2) parents and caregivers asking about the presence of unsecured firearms in other homes their children visit and play^¶¶^ as strategies to prevent unintentional firearm injuries and deaths. A recent study found that among firearm owners who reported not locking all firearms, almost one half believed locks were unnecessary or that a locked firearm might impede quick access in an emergency (*5*). This might partly explain why unsecured firearms in this study were most commonly accessed from a nightstand or other sleeping areas. Previous research found that parents often inaccurately predict their child’s knowledge of household firearm storage location and subsequent anticipated behavior if they should encounter a firearm (*6*,*7*). Additional findings from previous research indicate that many parents who own firearms incorrectly believe that their child can differentiate between a toy and a real firearm, and many trust that if their child encountered a real firearm, they would avoid it and tell an adult (*7*). In the current analysis, across all age groups, approximately two thirds of shooters were playing with or showing the firearm to another person when it discharged, and 10.6% of all children mistook the firearm for a toy (including approximately one quarter of those aged 0–5 years). These findings underscore the fact that parents’ reliance on children’s ability to distinguish between real and toy firearms and to not handle a firearm if they encountered one is insufficient to prevent unintentional firearm injury deaths of children.

Approximately three quarters of victims in this study were shot by another child, most commonly a friend, acquaintance, or sibling. Further, other children were present during or witnessed the fatal event in approximately one third of all incidents. Studies show that children exposed to firearm violence might experience poor mental health outcomes (e.g., anxiety) (*8*), further underscoring the importance of preventing unintentional firearm injury deaths among children and providing support for those involved in these incidents when they do occur.

When firearms are in the home, organizations have recommended storing them locked, unloaded, and separate from ammunition as effective strategies to prevent child firearm injuries and deaths.*** In addition, some states have enacted child access prevention (CAP) laws to hold firearm owners liable when a child gains access to an unsecured firearm.^†††^ While some research suggests CAP laws might be effective in preventing unintentional firearm injuries and deaths of children,^§§§^ other research has raised questions about awareness of the policies and the modest effects on secure firearm storage (*9*). Policymakers, health care professionals (including pediatricians), and others can partner with parents, caregivers, and firearm owners to better understand and address barriers to adoption of secure firearm storage practices.

### Limitations

The findings in this report are subject to at least four limitations. First, NVDRS data abstractors are limited to the information included in investigative reports, which might not include sufficient detail to identify all characteristics and circumstances for all decedents. In particular, information regarding firearm storage or access is often incomplete. Second, states and jurisdictions joined NVDRS in different years, so data were unavailable from all recipients for all years of this study, limiting the ability to capture all unintentional firearm injury deaths for the entire study period. Third, this report summarizes data from 49 states, the District of Columbia, and Puerto Rico (data for Florida are excluded because the data did not meet the completeness threshold for circumstances in NVDRS), thereby limiting the generalizability of these findings. Finally, unintentional firearm injury deaths might be misclassified on death certificates, leading to potential under- or over-ascertainment. However, NVDRS has been found to better identify these incidents than do death certificates alone (*10*).

### Implications for Public Health Practice

Unintentional firearm injury deaths of children are preventable. Secure firearm storage practices (e.g., firearm stored locked, unloaded, and separate from ammunition) have been identified as protective factors against unintentional and intentional (i.e., suicides) firearm injuries and deaths of children (*1*), highlighting the important role of policymakers, health care professionals and others in partnering with parents, caregivers, and firearm owners to promote secure firearm storage.

## Footnotes

* https://wisqars.cdc.gov/fatal-leading

† Data from 49 states, District of Columbia, and Puerto Rico that reported information to NVDRS during 2003–2021 were included in this analysis. These jurisdictions included Alaska, Maryland, Massachusetts, New Jersey, Oregon, South Carolina, and Virginia (2003–2021); Colorado, Georgia, North Carolina, Oklahoma, Rhode Island, and Wisconsin (2004–2021); Kentucky, New Mexico, and Utah (2005–2021); Ohio (2011–2021); Michigan (2014–2021); New York (2015–2018 and 2020–2021); Hawaii (2015, 2016, and 2019); Arizona, Connecticut, Kansas, Maine, Minnesota, New Hampshire, and Vermont (2015–2021); Illinois, Indiana, Iowa, Pennsylvania, and Washington (2016–2021); California, Delaware, District of Columbia, Nevada, Puerto Rico, and West Virginia (2017–2021); Alabama, Louisiana, Missouri, and Nebraska (2018–2021); Montana, North Dakota, and Wyoming (2019–2021); and Arkansas, Idaho, Mississippi, South Dakota, Tennessee, and Texas (2021).

§
https://www.cdc.gov/violenceprevention/pdf/nvdrs/nvdrsCodingManual.pdf

¶ Excludes nonpowder guns such as BB or pellet guns and air or gas rifles. The NVDRS case definition for unintentional firearm deaths corresponds to *International Classification of Diseases, Tenth Revision* underlying cause of death codes W32, W33, W34, and Y86. https://www.cdc.gov/violenceprevention/pdf/nvdrs/nvdrsCodingManual.pdf

**
https://wisqars.cdc.gov/fatal-reports

†† 45 C.F.R. part 46; 21 C.F.R. part 56; 42 U.S.C. Sect. 241 (d), 5 U.S.C. Sect. 552a, 44 U.S.C Sect. 3501 et seq.

§§ The final sample of included cases comprised 93.4% (1,179) of unintentional firearm injury deaths as identified by NVDRS; 5.9% (74) undetermined intent deaths by firearm; and 0.7% (nine) homicides with *International Classification of Diseases, Tenth Revision* codes W32, W33, W34, and Y86.

¶¶
https://www.healthychildren.org/English/safety-prevention/at-home/Pages/Handguns-in-the-Home.aspx

***
https://www.cdc.gov/violenceprevention/firearms/fastfact.html

†††
https://everytownresearch.org/rankings/law/secure-storage-or-child-access-prevention-required/

§§§
https://www.rand.org/research/gun-policy/analysis/child-access-prevention/unintentional-injuries.html
